# Determinants of Soil Microarthropod Community Biodiversity in the Svalbard High Arctic: Locality or Vegetation Community?

**DOI:** 10.1002/ece3.73440

**Published:** 2026-05-20

**Authors:** Stanisław Seniczak, Anna Seniczak, Sławomir Kaczmarek, J. Carlos Iturrondobeitia, Tomasz Marquardt, Stephen J. Coulson

**Affiliations:** ^1^ Department of Evolutionary Biology, Faculty of Biological Sciences Kazimierz Wielki University Bydgoszcz Poland; ^2^ Faculty of Applied Ecology, Agricultural Sciences and Biotechnology University of Inland Norway Hamar Norway; ^3^ Department of Zoology and Cellular Animal Biology University of the Basque Country Leioa Bizkaia Spain; ^4^ Department of Arctic Biology University Centre in Svalbard Longyearbyen Svalbard Norway

**Keywords:** Arctic, biodiversity, macroecology, soil microarthropod community, vegetation community, vegetation maps

## Abstract

Arctic ecosystems, such as those in Svalbard, are facing diverse stressors, being at the forefront of global warming while also facing increasing anthropogenic impacts. This poses a variety of threats to biodiversity, including soil microarthropod communities, which play a crucial role in the functioning of the Arctic ecosystems. Monitoring these groups in Svalbard is challenging due to limited baseline knowledge, logistically demanding and costly sampling, and the difficult and time‐consuming identification of soil microarthropod communities. In contrast, vegetation maps are relatively fast and easy to construct, often relying on remote sensing, and there are comprehensive vegetation maps available. These vegetation maps integrate numerous environmental characteristics into one vegetation community describing vegetation biodiversity. While relationships between soil microarthropod communities and vegetation communities have been documented in other regions, it remains unclear whether such patterns hold in the Arctic and whether existing vegetation maps could serve as proxies for soil microarthropod community composition and macroecology analyses. To address this question, we analyzed 172 soil samples collected from 33 localities across latitudinal and longitudinal gradients, representing 11 vegetation classes. In total, nearly 180,000 microarthropods were recorded, including approximately 63,000 Acari (87% Oribatida, 2% Mesostigmata, 10% Trombidiformes) and 116,000 Collembola. Oribatida and Mesostigmata were identified to family, while other groups were counted. Our analysis reveals that location is more important to soil microarthropod communities than vegetation community per se, indicating that these groups respond differently to local conditions. The variability of the Acari family abundances was mostly explained by Sørkapp, the southernmost and climatically harsh locality, followed by Fjortendejulibukta (northwestern Svalbard), which has a relatively mild climate but is subject to human disturbance. Locality also affected Oribatida diversity, mainly explained by Tjuvfjord in eastern Svalbard. At a larger scale, the approach of vegetation community classifications proved to be only weak proxies for the Acari and Collembola communities.

## Graphical abstract

Arctic ecosystems like Svalbard are rapidly changing, yet soil microarthropods remain difficult to monitor due to limited baseline data and demanding field and identification work. Using 172 soil samples from 33 localities, we tested whether vegetation maps could serve as proxies for soil microarthropod communities. Our results show that locality, rather than vegetation type, best explains community patterns, indicating that vegetation maps are weak predictors of Acari and Collembola communities.

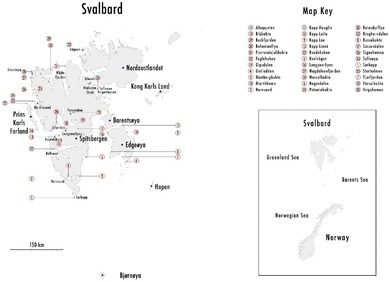

## 1 Introduction

Arctic ecosystems, such as those in the Svalbard archipelago, are at the forefront of global warming and environmental change which are posing significant threats to biodiversity, including soil microarthropod communities (Coulson et al. 2025). Svalbard is one of the regions with the fastest temperature increase on the globe, with a warming rate corresponding to 2.5 times the regional Arctic warming mean and 5 to 7 times the global warming mean (Isaksen et al. 2022). The effects of rising temperatures may differ across the Svalbard archipelago, which is among the most climatologically diverse regions globally due to the influence of ocean currents and air masses with contrasting thermal characteristics (Lapointe et al. 2024).

The relationship between soil microarthropods and vegetation communities is well documented in several regions outside the Arctic, including larger geographical areas, in forests (Erdmann et al. 2012; Liu and Wu 2024), alpine habitats (Ľuptáčik et al. 2021; Zhou et al. 2022), peatlands (Seniczak, Seniczak, Iturrondobeitia, et al. 2020), heather moorland (Nielsen et al. 2010, 2012), grasslands (Sabais et al. 2011), and industrially contaminated sites (Frouz et al. 2008). Some studies also address Arctic tundra, but these have been conducted at local scales (Coulson et al. 2003). This raises the question of whether such relationships hold in the Arctic and with the characteristic microarthropod community, and whether existing vegetation maps can serve as proxies for predicting soil microarthropod communities across pan‐Arctic scales and macroecology studies.

Despite the critical role of soil microarthropods in Arctic ecosystem functioning, their communities and species composition remain poorly understood (Gillespie et al. 2020) and is a targeted research priority highlighted by the Conservation of Arctic Fauna and Flora (CAFF) biodiversity working group of the Arctic Council (Aronsson et al. 2021). This knowledge gap is driven by several factors, with the logistical challenges and high costs of sampling in Arctic environments being among the most significant. As a result of uneven sampling in Svalbard, large areas lack baseline data on soil microarthropods. Furthermore, describing these communities requires considerable taxonomic expertise and is highly time‐consuming (Coulson et al. 2025). Environmental DNA (eDNA) and similar strategies are being developed but yet remain to be employed in many polar regions and, with regard to Svalbard, 50% of the species currently documented from the archipelago lack COI (cytochrome c oxidase subunit 1) barcode reference sequences in the Barcode of Life Data Systems database (Coulson et al. 2024). This contrasts markedly with the mapping of Arctic vegetation (Walker et al. 2005; Raynolds et al. 2024; CAVM 2025), where detailed vegetation community maps exist and are regularly updated using remote sensing techniques (Orndahl et al. 2025). Vegetation communities respond to local biotic and abiotic conditions; therefore, vegetation maps integrate numerous environmental characteristics into a single representation of community composition. Since soil microarthropod communities are dependent on the local environmental conditions, vegetation maps may therefore provide a reliable proxy for soil microarthropod communities if a firm relationship can be detected.

Svalbard provides a suitable location to test this hypothesis for the Arctic. Svalbard has a diverse invertebrate fauna comprised of over 1000 terrestrial and freshwater species (Coulson et al. 2024) and is regarded as having one of the most complete inventories for a location in the Arctic (Hodkinson 2013; Gillespie et al. 2020; Coulson et al. 2025). Moreover, a wide range of vegetation communities have been accurately mapped throughout a geographically wide archipelago with diverse and characteristic environments. The most abundant, diverse, and ecologically significant soil microarthropods in Svalbard are springtails (Collembola) and mites (Acari), which together account for roughly one quarter of all invertebrate species recorded in the archipelago (Coulson et al. 2024). Collembola are represented by 67 species across 10 families (Coulson et al. 2024) and play key roles in decomposition processes and soil microstructure formation (Rusek 1998). Similarly, oribatid mites, primarily saprophagous, contribute to the decomposition of organic matter (Walter and Proctor 2013). Oribatida have the richest alpha biodiversity among soil microarthropods in Svalbard, with 95 species and 30 families documented (Seniczak and Seniczak 2020; Seniczak, Seniczak, Schwarzfeld, et al. 2020; Coulson et al. 2024). The mite order Mesostigmata consists primarily of predators and is represented in Svalbard by 38 species across 13 families (Seniczak, Seniczak, Schwarzfeld, et al. 2020). Another order, Trombidiformes, encompasses diverse feeding groups—including algivores, bacterivores, fungivores, predators, and parasites (Walter and Proctor 2013)—but in Svalbard it is represented only by the suborder Prostigmata, comprising 15 terrestrial families and 30 documented species (Seniczak, Seniczak, Schwarzfeld, et al. 2020). Saprophagous microarthropods, mainly Collembola and Oribatida, play a crucial role in Svalbard ecosystems. In the absence of key soil macrofauna (e.g., earthworms, millipedes, woodlice), they serve as the primary consumers of soil organic matter, fragmenting and mixing it with other components of the organic layer and thereby increasing its availability to microbial decomposers (Hodkinson 2013).

To address the question of whether vegetation community maps can be used to predict soil microarthropod communities in Arctic regions we analyzed samples from the University Centre in Svalbard (UNIS) collection, gathered over 6 years from various parts of the archipelago. These samples span latitudinal and longitudinal gradients and represent 11 vegetation classes. We focused on the most abundant microarthropod groups: Acari (suborder Oribatida, orders Mesostigmata and Trombidiformes) and Collembola. Based on findings from other regions, we hypothesized that, at broad geographic scales in Svalbard: (i) vegetation and locality act as primary high‐level proxy variables determining microarthropod communities, and (ii) vegetation classification can be used to predict soil microarthropod community composition.

## 2 Material and Methods

### 2.1 Study Sites and Sampling

**FIGURE 1:**
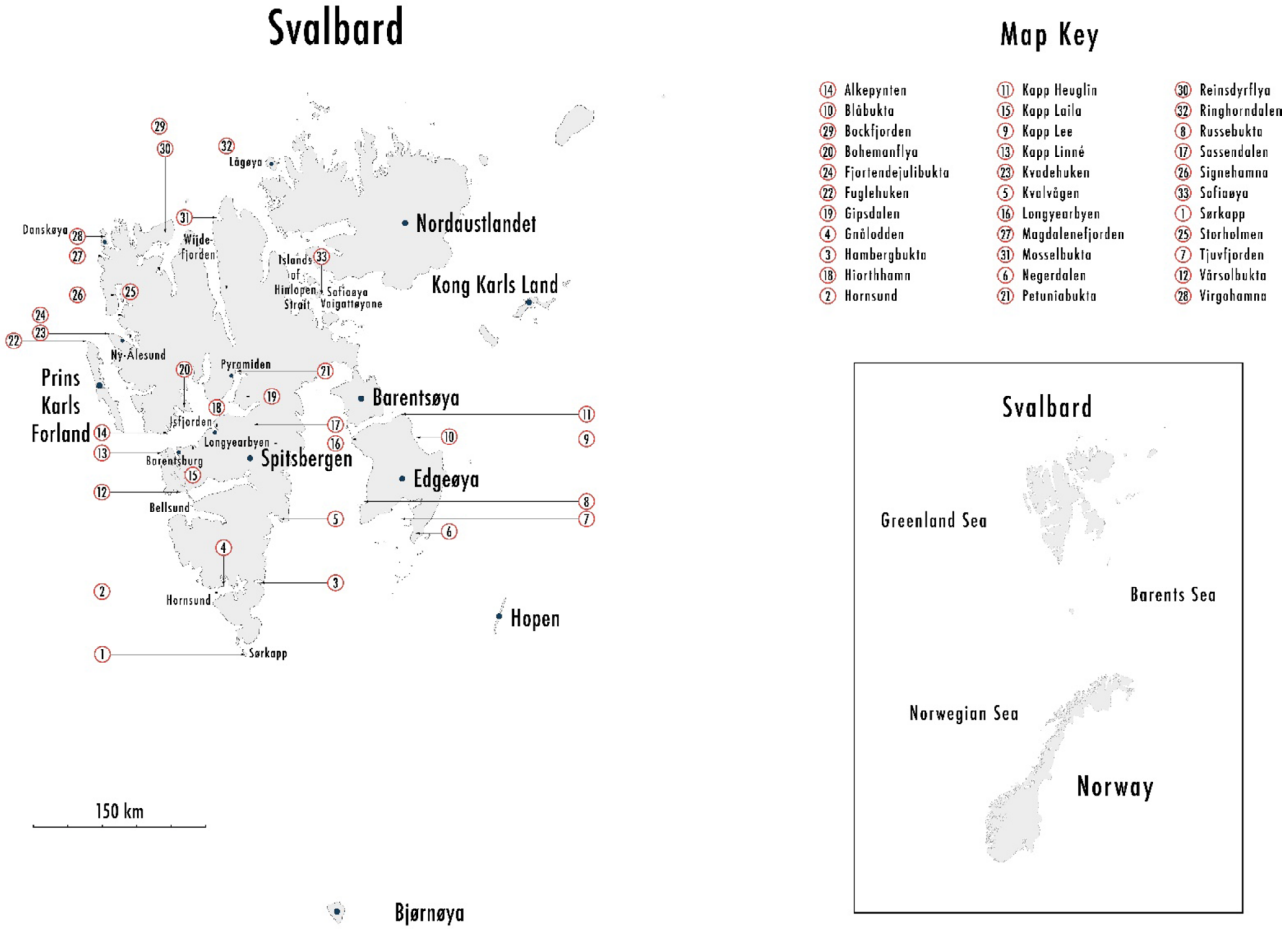
Locality of the study sites in Svalbard archipelago.

We used soil microarthropod extractions from the UNIS collection. These extractions came from soil samples collected by Stephen J. Coulson during the polar summer (a period of around 6 weeks from late June to early August) between 2009 and 2014 from 33 localities (Figure 1, Table 1) from coastal locations on southern, central, and northern parts of Spitsbergen as well as Danskeøya, Sofiaøya, and Edgeøya. These sampling sites were selected so as to include the principal vegetation communities of Svalbard and a variety of latitudinal and longitudinal locations within the archipelago. The majority of the samples were collected during various bachelor and postgraduate courses at UNIS and demonstrate the ability of field‐based education to produce publishable data. These sampling sites comprised 11 vegetation classes according to the revised Svalbard vegetation map (Johansen et al. 2012). The vegetation classes and their abbreviations, used later in the text, are listed in Table 1. The characteristics of these vegetation classes were given earlier (Seniczak, Seniczak, Graczyk, et al. 2017).

**TABLE 1:** Sampling design and characteristics of Oribatida (O) and Mesostigmata (M) communities: participation of juvenile stages (Juv, %), Shannon index (*H*), and number of families (*S*) in localities in Svalbard.

Locality (abbreviation)	No. samples	Vegetation class (abbreviation)	O			M			O + M
			Juv	*H*	*S*	Juv	*H*	*S*	*H*
Fjortendejulibukta (Fjor)	7	Arctic meadows (AM)	28	1.59	7	56	0.53	3	1.66
Fuglehuken (Fugl)	6		2	0.60	5	53	0.69	2	0.67
Reinsdyrflya (Rein)	6		25	1.20	9	44	0.57	2	1.28
Signehamn (Sign)	3		5	0.84	9	43	0.55	2	1.11
Storholmen (Stor)	6		46	1.70	9	23	1.07	3	1.76
Gipsdalen (Gips)	6	*Dryas*/*Carex* (DC)	53	1.16	5	29	0.49	2	1.24
Kapp Linné (KLin)	2		23	1.02	7	50	0.69	2	1.07
Magdalenefjorden (Magd)	3	Dry‐grass (DG)	17	0.05	5	100	0.00	1	0.30
Longyearbyen (Long)	7		49	0.76	4	0	0.00	1	0.78
Kvadehuken (Kvad)	2	*Dryas* heaths (DH)	34	1.29	4	—	—	0	1.29
Petuniabukta (Petn)	15		38	1.93	10	38	0.90	5	2.03
Mosselbukta (Moss)	5		17	1.56	11	70	0.68	2	1.62
Ringhorndalen (Ring)	9		31	1.52	10	39	0.31	3	1.77
Sørkap (Sørk)	12		18	1.17	10	100	0.38	2	1.19
Tjuvfjorden (Tjuv)	6		33	1.75	8	37	0.90	3	1.84
Danskeøya (Dans)	3	Gravel barren (GB)	2	0.74	4	39	0.00	1	1.01
Blåbukta (Blåb)	1	*Luzula* vegetation (LV)	83	0.30	3	—	—	0	0.30
Bockfjorden (Bock)	8		26	1.89	11	35	0.44	2	2.04
Hambergbukta (Hamb)	3		37	0.76	4	100	0.00	1	0.87
Negerdalen (Negr)	2		50	0.52	4	—	—	0	0.52
Sofiaøya (Sofi)	2	Moderate snowbed (MS)	36	1.42	5	39	1.00	4	1.61
Alkepynten (Alkp)	3	Moss tundra (MT)	29	0.21	4	0	0.00	1	0.22
Kapp Laila (KLai)	10		17	0.61	6	47	0.42	2	0.73
Kapp Lee (KLee)	4	Pioneer vegetation (PV)	78	0.68	7	7	0.50	2	0.86
Hiorthamn (Hior)	6	Rich moss tundra and bird cliff vegetation (RT)	13	1.26	10	88	0.98	4	1.33
Hornsund (Horn)	2		22	0.77	6	—	—	0	0.77
Kapp Heuglin (KHeg)	1		11	0.54	3	10	0.00	1	0.58
Kvalvågen (Kval)	3		20	0.61	7	—	—	0	0.61
Russebukta (Russ)	12		26	1.84	9	20	0.50	4	1.94
Vårsolbukta (Vårs)	3		15	0.37	7	41	0.68	2	0.41
Bohemanflya (Bohm)	2	Swamps (SW)	17	0.73	6	86	0.00	1	0.84
Gnålodden (Gnål)	4		20	1.44	6	13	0.69	2	1.50
Sassendalen (Sass)	8		27	1.48	7	76	0.78	4	1.55

Samples were collected mainly from coastal sites usually between 500 m to 1000 m from the shoreline and through the organic soil where possible. The exception to this being the vegetated slope in Ringhorndalen (about 100 m asl and 4 km from the coast). The sites are therefore typical of the range of vegetation communities present in Svalbard. Soil samples (soil and plant cover) were collected from the typical vegetation communities at each locality. In total, 172 samples were collected, and the number of samples from each locality varied between 1 and 15 due to an opportunistic sampling strategy and from each vegetation class between 2 and 49. Each sample had dimensions of c. 10 × 10 cm and 5 cm deep (the usual maximum depth of the organic soil in these regions). The samples were kept cool until being extracted in Tullgren funnels (Burkard Scientific Ltd., Uxbridge, UK) at UNIS usually within 5 days of collection. Samples were extracted until the upper surface was completely dry, usually taking 3–4 days. Extracted animals were preserved in 96% ethanol.

### 2.2 Microarthropod Analyses

Microarthropods were sorted under stereomicroscope into groups (Acari, that were sorted to Oribatida, Mesostigmata, and Trombidiformes, and Collembola), including adults and juveniles, and counted. Due to very abundant material (nearly 180,000 specimens) only Oribatida and Mesostigmata were identified to family level, while other groups were counted. Lower taxonomy resolution has been argued to be sufficient in ecological studies (e.g., Ellis 1985; Terlizzi et al. 2003), including family approach in Oribatida and Mesostigmata (e.g., Seniczak et al. 2018; Meehana et al. 2019). Adult Oribatida were identified to families using Behan‐Pelletier and Lindo (2022), while juveniles were identified according to various publications (Grandjean 1963; Behan‐Pelletier 1985; Seniczak and Seniczak 2010, 2012; Pfingstl and Krisper 2011; Ermilov et al. 2012; Seniczak et al. 2015; Seniczak et al. 2017a, 2017b; Seniczak, Seniczak, Graczyk, et al. 2017; Seniczak, Ivan, et al. 2021; Seniczak, Seniczak, Kaczmarek, et al. 2022; Norton and Ermilov 2022). Identification of Mesostigmata was based on the following, Sellnick (1958); Hyatt (1980); Karg (1993); Makarova (2000); Gwiazdowicz et al. (2011a, 2011b); Kolodochka and Gwiazdowicz (2014); and Teodorowicz et al. (2014).

### 2.3 Statistical Analyses

#### 2.3.1 Indices

Microarthropod communities were characterized with abundance per 500 cm^3^; mean values and standard deviation were calculated in Microsoft Excel 365. Oribatida and Mesostigmata communities were additionally characterized with Shannon diversity index (*H*), Evenness index, number of families (*S*), and percentage of juvenile instars. The abundance of families of Oribatida and Mesostigmata is given.

#### 2.3.2 Multivariate Analysis

Multivariate analysis was performed with CANOCO 5.0 (Microcomputer Power, Ithaca, NY, USA; Ter Braak 1988; Ter Braak et al. 1995). Dependent variables (continuous quantitative) were abundances of Oribatida (juveniles, adults, total), Mesostigmata (juveniles, adults, total), Trombidiformes (total), all Acari (total), Collembola (total), and microarthropods, Shannon index of Oribatida, Mesostigmata, Oribatida + Mesostigmata. Independent variables were 33 localities and 11 vegetation classes (Table 1), and coordinates (i.e., latitude and longitude of each locality).

First, constrained canonical correspondence analysis with a forward selection (CCA FS) was carried out to evaluate which factors, locality or vegetation classes, contribute most to the variability of the data. Next, the variation partitioning between locality and vegetation was assessed to determine the degree that explained the variability of the data (abundance of Oribatida and Mesostigmata families). This was followed by a Principal Coordinate Analysis of Neighbor Matrices (PCNM) to test for spatial structure (based on geographical coordinates of the localities) of the microarthropod communities manifested as a spatial correlation among the sampling localities. The PCNM allows quantification of the importance of the independent variables tested and whether there are interdependencies between them.

A constrained redundancy analysis (RDA) was subsequently applied to examine if locality/vegetation affects the abundance of all microarthropods, Collembola, total abundance of Acari, Oribatida, Mesostigmata (including adults and juveniles of both groups), and Trombidiformes, and diversity indices (number of families, Shannon and Evenness indices) of Oribatida, Mesostigmata, and Oribatida and Mesostigmata together.

## 3 Results

### 3.1 The Abundance and Diversity Indices

**FIGURE 2:**
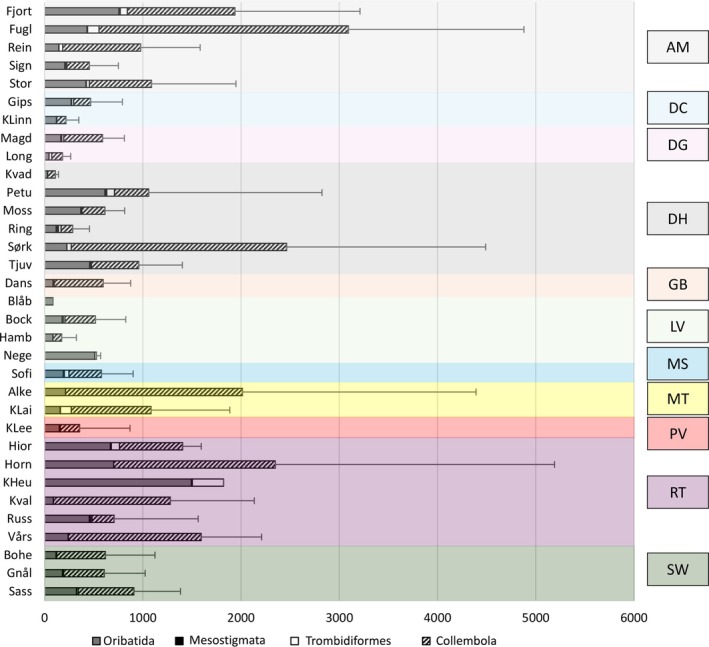
Mean abundance of microarthropods (bars) and standard deviation (error bars) in localities in Svalbard; different vegetation classes are marked in different colors, abbreviations in Table 1.

In total, 179,386 microarthropods were identified, including 63,323 Acari (55,279 Oribatida, 1455 Mesostigmata and 6589 Trombidiformes) and 116,063 Collembola. The highest abundance of microarthropods was found at Fuglehuken (over 3000 specimens per 500 cm^3^) and the lowest was at Blåbukta (86 specimens per 500 cm^3^) (Figure 2). At most localities, the Collembola were more abundant than the Acari, but at some sites they were fewer than mites or even absent (Blåbukta and Negerdalen with LV vegetation class and Kapp Heuglin with RT). Oribatida were always the most abundant group of mites and present at all study sites (Figure 2). Other Acari groups were absent at some localities, for example, Mesostigmata were absent from five study sites. The highest abundance of Oribatida and Trombidiformes was at Kapp Heuglin and of Mesostigmata, in Ringhorndalen. The abundance of Collembola was greatest at Fuglehuken. In different vegetation classes a large variation of microarthropod abundance was noted, but the highest mean abundance of microarthropods, and among them of the springtails, was observed in AM. The Acari achieved greatest mean abundances in RT but were also abundant in AM.

In total, 15 families of Oribatida and seven families of Mesostigmata occurred. The highest diversity of Oribatida was recorded in Petuniabukta, and the lowest was in Magdalenefjord. The highest diversity of Mesostigmata was at Storholmen, while at seven sites this group was represented by only one family and the diversity index could not be calculated. The diversity of families of Oribatida and Mesostigmata combined was highest in Bockfjord, and the lowest in Alkepynten (Table 1).

The most abundant oribatid family was the Oppiidae (28%), followed by Brachychthoniidae (23%). The most abundant mesostigmatid family was the Zerconidae (66%), followed by the Ascidae (29%). In some oribatid families the juveniles were more abundant than adults (Crotoniidae, Ceratozetidae, Eulohmanniidae, Hermanniidae, Oribatulidae, Peloppiidae); in other families the juveniles were less abundant than adults or absent (Damaeidae, Tegoribatidae, Trhypochthoniidae). In most families of the Mesostigmata the adults were more abundant than juveniles, and in Veigaiidae only adults were found (Data S1). Only in Parasitidae were the juveniles more abundant than adults, and in Halolaelapidae only juveniles were found.

### 3.2 The Effect of Environmental Variables on the Microarthropod Communities

**FIGURE 3:**
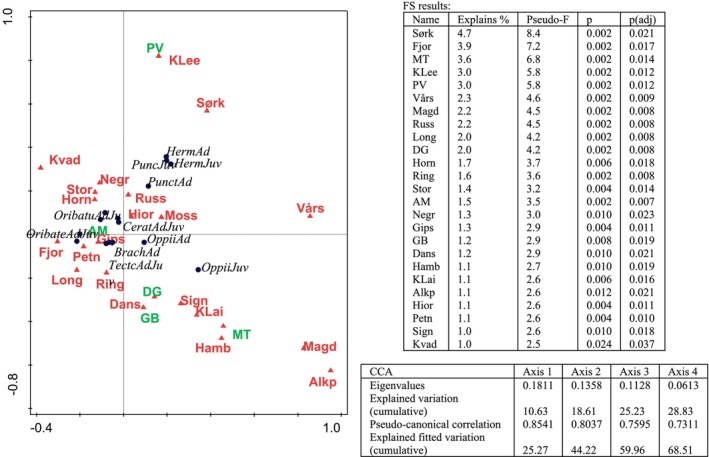
Canonical correspondence analysis (CCA) represented by 15 best fitted Acari families (circles) and most contributing environmental variables (triangles), green letters denote vegetation classes, red are localities and in Italics are taxa. Forward selection results of most contributing (*p* < 0.05) environmental variables are listed in the upper table associated with figure and CCA results are presented in the lower table. Abbreviations of vegetation classes and localities are given in Table 1. Ad, adults; Brach, Brachychthoniidae; Cerat, Ceratozetidae; Herm, Hermanniidae; Juv, juveniles; Oppii, Oppiidae; Oribate, Oribatellidae; Oribatu, Oribatulidae; Punc, Punctoribatidae.

The CCA FS (factors included in the analysis: localities and vegetation classes) explains 42.08% of the variability of the abundance of the Acari families, and adjusted explained variation of 32.95%, DF = 32. The factor that mostly explained this variability was Sørkapp, followed by Fjortendejulibukta. The family that mostly differentiated Sørkapp from the other localities was the Hermanniidae, represented both by adults and juveniles, while Fjortendejulibukta was distinguished by the Oribatellidae, also represented by adults and juveniles (Figure 3).

The factors mostly explaining the variability of the mite communities are presented in Figure 3 (20 localities and 5 vegetation classes). The other non‐significant factors (*p* > 0.05) (13 localities and 6 vegetation classes) were excluded from the analyses with FS.

The results of the variation partitioning showed that vegetation alone does not explain abundances of communities but has an explanatory value when included in the variation of sample locality. When vegetation (a) was considered together with locality (b) and shared part (c) (a + b + c) it was significant for explaining variation of the abundance of the Acari families (*F* = 3.8, *p* = 0.002), but vegetation alone was not significant, and locality alone was significant (*F* = 3.6, *p* = 0.002).

The spatial component alone (b) (locality factor and shared part removed) does not play any important role in determining microarthropod abundances (PCNM), but spatial variability is within environmental factor locality. Locality factor alone (a), with shared part (c) and spatial (b) removed, explains 11.7%. Significance test for locality (a), considered together with spatial variable (b) and shared part (c), showed that together (a + b + c) they were significant for explaining the 34.95% in the variation of the abundance of the Acari families (*F* = 3.7, *p* = 0.002). Also, a + c (locality+shared) was significant (*F* = 3.8, *p* = 0.002, 34.8%), and b + c (space+shared) was significant (*F* = 4.0, *p* = 0.002, 23.2%).

**FIGURE 4:**
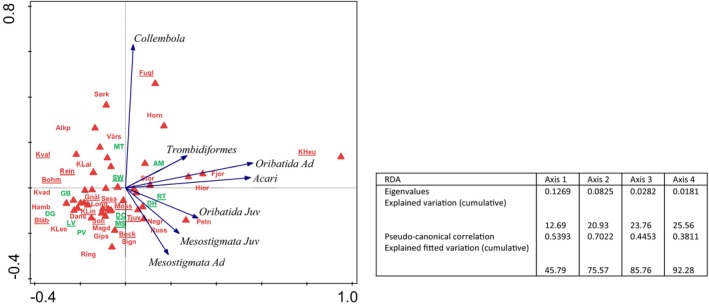
Results of redundancy analysis (RDA) of abundances of microarthropod groups vs localities (red triangles and red letters) and vegetation factors (green letters), RDA results are presented in the table associated with figure. Abbreviations in Table 1. Insignificant factors are underlined.

Redundancy analysis indicated that locality was significant for the abundance of all microarthropods (adjusted explained variation: 11.06%, permutation test results on first axis: pseudo‐*F* = 0.6, *p* = 0.131, on all axes: pseudo‐*F* = 1.7, *p* = 0.026, DF = 32) (Figure 4). Locality was also significant for the total abundance of Acari (adjusted explained variation: 15.37%, permutation test results on first axis: pseudo‐*F* = 2.0, *p* = 0.036, on all axes: pseudo‐*F* = 2.0, *p* = 0.036, DF = 32) and Collembola (adjusted explained variation: 31.81%, permutation test results on first axis: pseudo‐*F* = 3.5, *p* = 0.002, on all axes: pseudo‐*F* = 3.5, *p* = 0.002, DF = 32). For the Acari groups, the results were approaching significance for Oribatida (adjusted explained variation: 13.57%, permutation test results on first axis: pseudo‐*F* = 1.8, *p* = 0.054, on all axes: pseudo‐*F* = 1.8, *p* = 0.054, DF = 32), but insignificant for Mesostigmata and Trombidiformes. The locality was significant for the abundance of adult Oribatida (adjusted explained variation: 20.58%, permutation test results on first axis: pseudo‐*F* = 2.4, *p* = 0.011, on all axes: pseudo‐*F* = 2.4, *p* = 0.011, DF = 32), but not for juvenile Oribatida, or adult and juvenile Mesostigmata.

The locality affected the Shannon diversity index of Oribatida (adjusted explained variation: 45.51%, permutation test results on first axis: pseudo‐*F* = 5.4, *p* = 0.001, on all axes: pseudo‐*F* = 5.4, *p* = 0.001, DF = 32), and of Oribatida and Mesostigmata considered together (adjusted explained variation: 43.62%, permutation test results on first axis: pseudo‐*F* = 5.1, *p* = 0.001, on all axes: pseudo‐*F* = 5.1, *p* = 0.001, DF = 32), but not of Mesostigmata alone. All these analyses but with the vegetation factor added gave the same results since vegetation alone was not significant.

**FIGURE 5:**
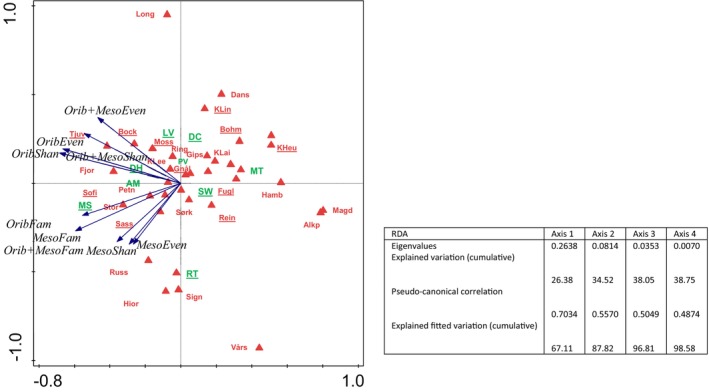
Redundancy analysis (RDA) of diversity indices (Fam—number of families, Shan—Shannon index, Even—Evenness index) of Oribatida (Orib) and Mesostigmata (Meso) communities vs localities (red triangles and red letters) and vegetation classes (green letters), RDA results are presented in the table associated with figure. Abbreviations in Table 1. Insignificant factors are underlined.

The results of redundancy analysis of diversity indices (number of families, Shannon and Evenness indices of Oribatida, Mesostigmata, and Oribatida + Mesostigmata) show two differing responses: one for Shannon index and Evenness index of Oribatida and Oribatida + Mesostigmata, and another for the number of families of Oribatida and Oribatida + Mesostigmata, and Shannon and Evenness indices of Mesostigmata (Figure 5).

## 4 Discussion

The locality itself explains much of the variability in microarthropod communities contrary to expected vegetation community. Locality encompasses not only the vegetation community variability but also the spatial arrangement of sampling sites. These two components only manifest themselves clearly when they are treated without the locality factor. In such cases, 24% of the variation is attributed to spatial factors (i.e., latitude and longitude in the dataset), while only 11% is explained by vegetation type. Despite this, it should be kept in mind that locality also includes other factors such as historic stochastic colonization events or idiosyncratic succession processes that, together, combine to create the microarthropod communities observed in the present. Nonetheless, and while locality encompasses other various determining factors that cannot be untangled here, locality—rather than vegetation or spatial arrangement—emerges as the most important determinant of microarthropod communities at a larger scale in the Svalbard archipelago. Our first hypothesis, which proposed that vegetation and locality would function as overarching proxy variables structuring these communities, is therefore only partially supported. Consequently, our second hypothesis—that vegetation classification can reliably predict soil microarthropod community composition—must be rejected.

The absence of a single dominant driver at a larger spatial scale aligns with previous findings that climate, together with impacts such as nitrogen deposition, drives changes in biodiversity and ecosystem functioning at broad scales (Sala et al. 2000; Mitchell et al. 2016). In contrast, at local scales, factors such as habitat quality, microclimate, and food availability and quality become more influential (Mitchell et al. 2016). Consequently, earlier studies in Svalbard that focused on relatively small areas near Ny‐Ålesund demonstrated that plant species may serve as useful proxy indicators for soil microarthropod communities at local scales (Coulson et al. 2003) but fail to capture a broader geographic macroecology perspective. Our samples were collected from multiple regions across the Svalbard archipelago, including coastal sites in the western, eastern, northern, and southern areas. Locality emerged as a significant factor influencing microarthropod abundance, including the total abundance of Acari and Collembola, as locality combines multiple stressors such as local climate, isolation, and vegetation community.

Studies conducted in areas of similar geographic size may yield different conclusions depending on the degree of environmental heterogeneity. For example, in forests located within a relatively uniform, mild climate, forest type was identified as the most important factor shaping Oribatida and Mesostigmata mite communities (Bolger et al. 2014; Corral‐Hernández et al. 2016; Seniczak, Niedbała, et al. 2021). However, when large regional climatic differences were present (Erdmann et al. 2012) or under severe climatic conditions (Kamczyc et al. 2023), locality became the primary driver of the Acari communities. Similarly, on sub‐Antarctic oceanic islands, abiotic variables such as temperature and moisture—rather than plant characteristics—were found to be the main determinants of community composition (Hugo‐Coetzee and Le Roux 2018). These strong effects of climatic forces raise concerns about the potential impacts of climate change on soil communities (Van der Putten et al. 2010; Ott et al. 2012).

As for the individual Acari groups, locality significantly affected the total abundance of Oribatida and adult Oribatida, but not juvenile Oribatida, Mesostigmata (adults and juveniles), or Trombidiformes. This pattern likely reflects the higher abundance of adult Oribatida compared to juveniles in most localities (except Blåbukta and Gipsdalen), as well as their dominance over Mesostigmata and Trombidiformes across all sites, which limited statistical significance for these latter groups. The low abundance of juvenile Oribatida was associated with the predominance of the family Oppiidae, whose juveniles exhibit poor extraction efficiency. These mites develop rapidly (Pfingstl and Schatz 2021) and may enter a quiescent phase during extraction, preventing them from leaving the sample (Seniczak 1978). However, in many other cases, juvenile stages of mites and other arthropods are more abundant than adults, with important ecological and biodiversity implications (Seniczak, Seniczak, Iturrondobeitia, et al. 2022; Caterino and Recuero 2024).

The variability of the Acari families was mostly explained by Sørkapp, the southernmost tip of Spitsbergen, which is influenced by cold sea currents and characterized by a high‐Arctic climate. This locality exhibited one of the highest microarthropod abundances, yet Oribatida family diversity was moderate, and Mesostigmata diversity was low. Sørkapp was distinguished by the presence of Hermanniidae, represented in Svalbard by two species— *Hermannia reticulata* Thorell, 1871 and *H. scabra* (L. Koch, 1879)—both primarily occurring in circumpolar regions (Seniczak, Seniczak, Schwarzfeld, et al. 2020) and typical of tundra habitats (Behan‐Pelletier and Lindo 2022). Sørkapp is particularly sensitive to climate change (Eidesen et al. 2018), which may have cascading effects on soil microarthropods, underscoring the need for focused monitoring in future studies (Ziaja 2006). Generally, Arctic microarthropods respond negatively to prolonged exposure to elevated temperatures (Christoffersen et al. 2024; Sørensen et al. 2024). However, some oribatid mites, including *H. reticulata* , appear tolerant to warming, as shown in temperature manipulation experiments (Coulson et al. 1996; Webb et al. 1998), suggesting that Hermanniidae may persist at Sørkapp despite rapid climate change.

Another locality that largely explained the variability in Acari family abundance was Fjortendejulibukta, which has a relatively mild climate due to the influence of the warm West Spitsbergen Current but faces anthropogenic pressure as a popular tourist destination. This locality was distinguished by the presence of Oribatellidae, represented in Svalbard by a single species, *Oribatella arctica* Thor, 1930. Described originally from Svalbard, *O. arctica* is a typical Arctic species known from Edgeøya and Spitsbergen (Seniczak, Seniczak, Schwarzfeld, et al. 2020) and prefers moist and shrub tundra habitats (Behan‐Pelletier 1998). In Svalbard, it was recorded in only six of 33 investigated localities, favoring Arctic meadows with rich vegetation, such as those below the Fjortendejulibukta bird cliffs, where it achieved a density of 300–98,000 individuals per m^2^ (Seniczak et al. 2015). The family occurred in all seven samples collected from this locality, comprising both adults and juveniles, with adults averaging 60%. Oribatellidae was also found in two other localities with dense *Dryas* heaths: in Petuniabukta, where it appeared in three samples with a mean abundance of 95 specimens per 500 cm^3^ (range: 0–980 specimens; adults 67%), and in Ringhorndalen, where only one adult specimen was recorded. Arctic species such as *O. arctica* deserve special attention in biodiversity conservation and environmental protection efforts in Svalbard.

Although vegetation was not significantly correlated with the abundance of any microarthropod group studied, the mean abundance of Acari was greatest in rich moss tundra and bird cliff vegetation. These habitats are enriched with nutrients from bird guano and characterized by lush vegetation and abundant and rich soil communities (Zmudczyńska‐Skarbek et al. 2017, 2024). Similarly, high mean abundances of Acari and the greatest abundance of Collembola were observed in Arctic meadows, which feature luxuriant vegetation (including bird cliff communities) and deep, thick organic soils that retain moisture—unlike the often thin organic or mineral soils elsewhere in Svalbard—creating favorable microhabitat conditions for soil fauna (Seniczak et al. 2017a). The large variation in soil microarthropod abundance among localities within the same vegetation class can be partly attributed to locality‐specific factors and the highly heterogeneous microscale environment typical of Arctic ecosystems (Jónsdóttir 2005).

The diversity indices of Oribatida and Oribatida combined with Mesostigmata were significantly influenced by locality, primarily explained by Tjuvfjord, located on Edgeøya in the eastern part of the Svalbard archipelago (Ávila‐Jiménez et al. 2019). Svalbard's biodiversity is strongly shaped by the two major sea currents and postglacial colonization routes, mainly from the East (Siberia) and the West (Canada and Greenland), whereas colonization from southern regions such as Scandinavia appears less common (Alsos et al. 2007; Brožová et al. 2023). Consequently, the eastern region, including Tjuvfjord, is expected to host communities distinct from those in the western part (Coulson et al. 2014; Ávila‐Jiménez et al. 2019).

The most abundant oribatid family in our study was Oppiidae, represented in Svalbard by nine species (Seniczak and Seniczak 2020; Seniczak, Seniczak, Schwarzfeld, et al. 2020), which feed primarily on fungi (Behan‐Pelletier and Lindo 2022). Some species, such as *Oppiella nova* (Oudemans, 1902), are considered highly eurytopic and act as pioneers in ecological succession (Skubała 2004). Another abundant family was Brachychthoniidae Thor, 1934, the richest oribatid mite family in Svalbard, with 15 species recorded (Coulson et al. 2024). Members of this family are small (150–335 μm) and are common and often dominant in Svalbard, as documented in Hornsund (Seniczak and Plichta 1978), Longyearbyen, and Petuniabukta (Seniczak et al. 2014). Brachychthoniidae feed on algae and fungi (Behan‐Pelletier and Lindo 2022), making them well adapted to the challenging conditions of polar regions.

Overall, the microarthropod community was found to depend primarily on locality rather than vegetation, latitude, or longitude. This result was surprising, as the soil microarthropod community is often assumed to consist mainly of generalists (Luxton 1972; Ponsard and Arditi 2000; Scheu and Falca 2000). In Svalbard, however, pronounced community differences between locations and a high degree of habitat specificity are observed, as previously hinted for with the Araneae (Dahl et al. 2018). Thus, the microarthropod community of the archipelago is not composed solely of Arctic generalists occurring throughout the region. On the contrary, the species assumed to be generalists show a reliance on environmental conditions not reflected in vegetation community composition per se. Moreover, observed microarthropod community composition may also reflect immigration history following the last glaciation, as demonstrated for the vegetation communities (Alsos et al. 2007; Brožová et al. 2023). The Svalbard fauna consists of species that colonized after the retreat of ice at the end of the Last Glacial Maximum (LGM), approximately 10,000 BP (Ingólfsson and Landvik 2013), and is therefore relatively young. There is little evidence that flora and fauna survived the LGM in situ (Coulson et al. 2014; Brožová et al. 2023). Although predicting soil microarthropod communities based on vegetation maps proved largely impossible, this study highlights substantial community differences between locations, strongly encouraging further sampling, particularly in regions of Svalbard that remain *terra incognita* with respect to soil fauna for example Edgeøya (Ávila‐Jiménez et al. 2019; Aronsson et al. 2021; Coulson et al. 2025).

## 5 Conclusions

This study provides the first evidence that, at a large spatial scale in the Arctic (Svalbard), microarthropod community composition depends primarily on locality rather than vegetation community or geographic coordinates (latitude and longitude). Consequently, vegetation maps fail to serve as reliable proxies for describing soil communities of Acari and Collembola at this scale since the strong environmental heterogeneity likely combined with distinct colonization routes following the last glacial maximum, colonization history and the peculiarities of individual community succession, conspire to mask any effect of vegetation community. This strongly highlights the need for further sampling, particularly in regions of Svalbard and the wider Arctic, that remain unexplored with respect to soil fauna as has been emphasized by previous authors (e.g., Aronsson et al. 2021).

## Author Contributions

**Stanisław Seniczak:** conceptualization (lead), data curation (equal), formal analysis (equal), investigation (equal), methodology (lead), resources (equal), validation (equal), writing – original draft (equal), writing – review and editing (equal). **Anna Seniczak:** conceptualization (equal), data curation (equal), formal analysis (equal), investigation (equal), methodology (equal), resources (equal), validation (equal), visualization (equal), writing – original draft (equal), writing – review and editing (equal). **Sławomir Kaczmarek:** conceptualization (equal), data curation (equal), investigation (equal), methodology (equal), resources (equal), validation (equal), writing – original draft (equal), writing – review and editing (equal). **J. Carlos Iturrondobeitia:** conceptualization (equal), formal analysis (lead), investigation (equal), methodology (equal), resources (equal), visualization (equal), writing – original draft (equal), writing – review and editing (equal). **Tomasz Marquardt:** conceptualization (equal), data curation (equal), investigation (equal), methodology (equal), resources (equal), validation (equal), writing – original draft (equal), writing – review and editing (equal). **Stephen J. Coulson:** conceptualization (equal), data curation (equal), investigation (equal), methodology (equal), resources (equal), validation (equal), writing – original draft (equal), writing – review and editing (equal).

## Funding

The authors have nothing to report.

## Conflicts of Interest

The authors declare no conflicts of interest.

## Supporting information


**Data S1:** ece373440‐sup‐0001‐Supinfo.xlsx.

## Data Availability

All the required data are uploaded as Supporting Information S1.
